# Supplementary material for: Platinum Black/Gold Nanoparticles/Polyaniline Modified Electrochemical Microneedle Sensors for Continuous In Vivo Monitoring of pH Value
Source: Polymers (Basel). 2023 Jun 23;15(13):2796. doi: 10.3390/polym15132796 (PMC10346494; doi:10.3390/polym15132796)
Supplement: Supplementary file 1 [file polymers-15-02796-s001.zip › polymers-2442452-supplementary.pdf]

Supporting Information

Platinum black/gold nanoparticles/polyaniline modified electrochemical microneedle sensors for continuous in vivo monitoring of pH value

Tao Ming[a], Tingting Lan[a], Mingxing Yu[a], Hong Wang[b], Juan Deng[b], Deling Kong[a], Shuang Yang[a*], Zhongyang Shen[a*]

[a] Research Institute of Transplant Medicine, Tianjin First Central Hospital, Nankai University, Tianjin 300190, China

[b] Institute of Biomedical Engineering, Chinese Academy of Medical Sciences & Peking Union Medical College, Tianjin 300192, China

[*]Correspondence: yangshuang@nankai.edu.cn (S. Yang), zhongyangshen@nankai.edu.cn (Z. Shen).

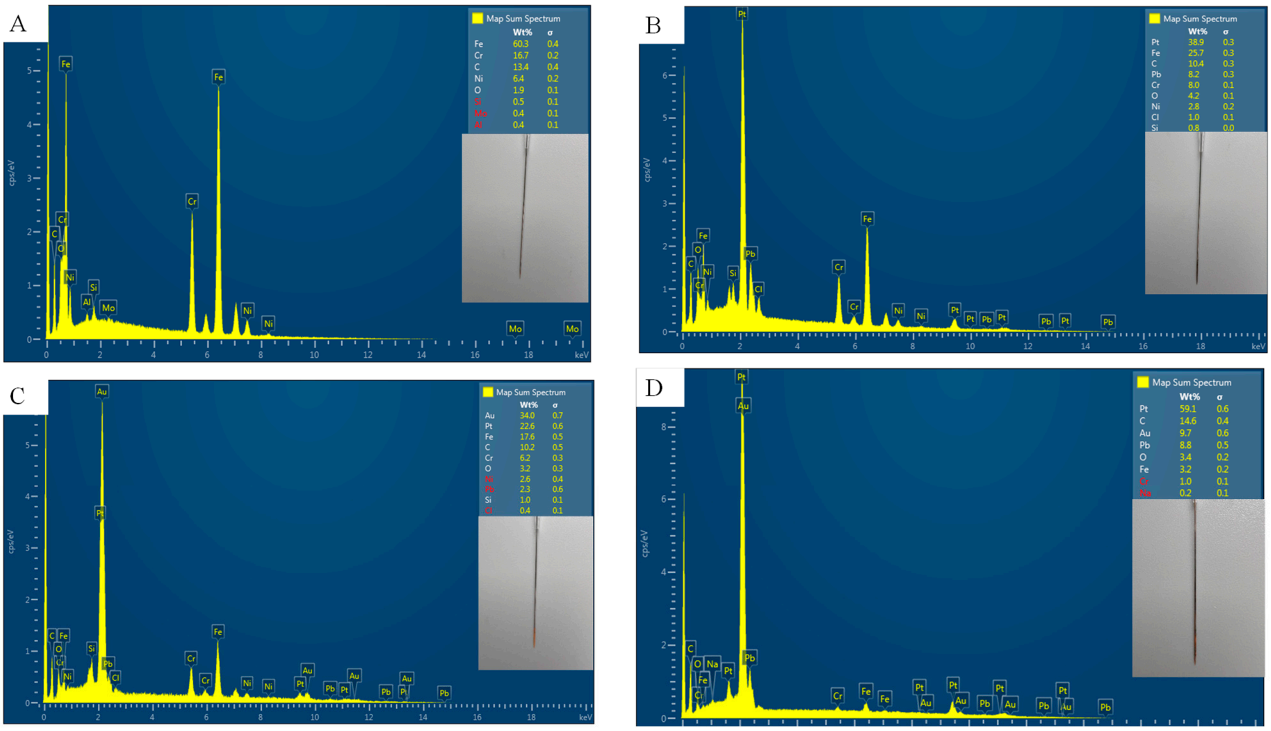

Fig. S1 The physical image and the EDS results of the electrode: (A) Bare AN; (B) Ptb/AN; (C) AuNPs/Ptb/AN; (D) PANI/ AuNPs/Ptb/AN;

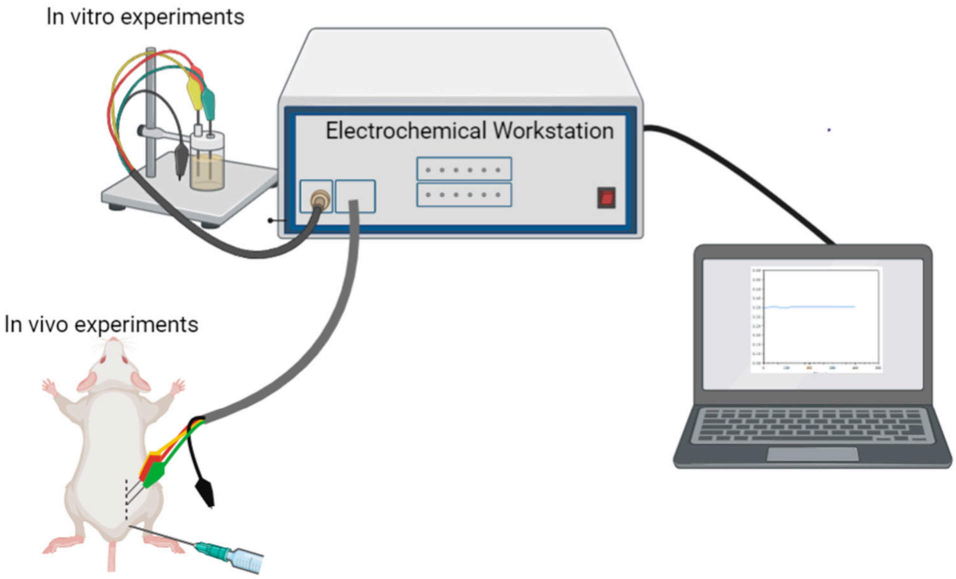

15

Fig. S2 Schematic of the measurement setup

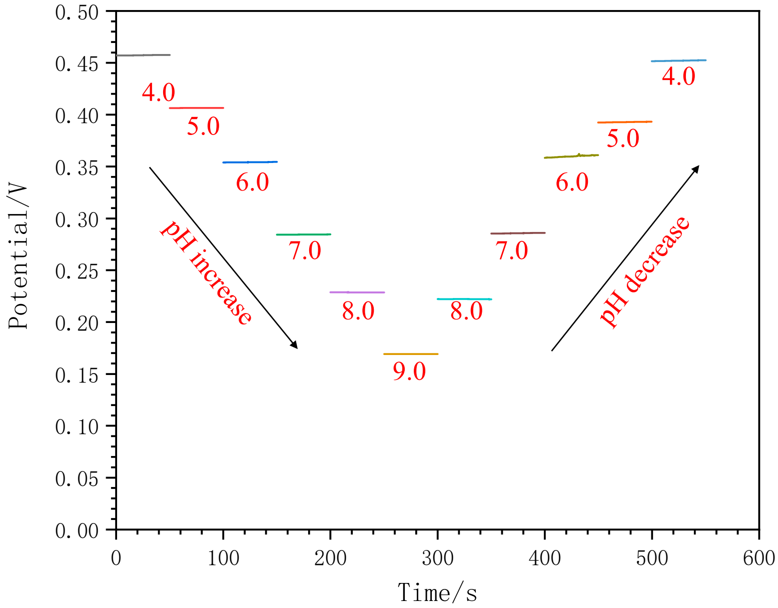

Fig. S3 The real-time OPCT responses in various pH buffer solutions.

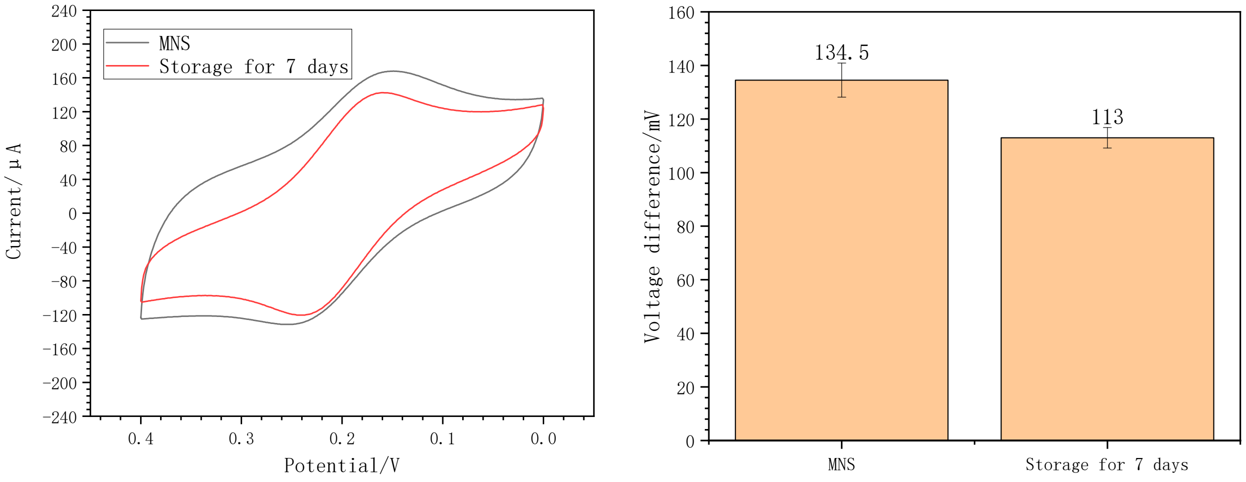

A   B

12

Fig. S4 The stability of the MNS. (A) The CV response of the proposed MNS and MNS after storing in standard serum for seven days; (B) The change in detection capability of the sensor;
